# DNA修复基因*XPD* G312A多态性与肺癌易感性关系的*meta*分析

**DOI:** 10.3779/j.issn.1009-3419.2010.05.27

**Published:** 2010-05-20

**Authors:** 朝蓉 梅, 文军 邓, 清华 周

**Affiliations:** 1 300052 天津，天津医科大学总医院，天津市肺癌研究所，天津市肺癌转移与肿瘤微环境重点实验室 Tianjin Key Laboratory of Lung Cancer Metastasis and Tumor Microenvironment, Tianjin Lung Cancer Institute, Tianjin Medical University General Hospital, Tianjin 300052, China; 2 610041 成都，四川大学华西医院电教中心 Audio-visual Center, West China Hospital of Sichuan University, Chengdu 610041, China

**Keywords:** XPD, 单核苷酸多态性, 肺肿瘤, *meta*分析, XPD protein, Single nucleotide polymorphism, Lung neoplasms, *meta*-analysis

## Abstract

**背景与目的:**

已有的研究结果显示DNA修复基因*XPD* G312A多态位点与肺癌发生存在相关性，但研究结果尚未有一致性结论。本研究旨在通过*meta*分析的方法，综合评价DNA修复基因XPD G312A多态位点与肺癌发病风险的相关性。

**方法:**

检索PUBMED、EMBASE、清华CNKI全文数据库、万方全文数据库中*XPD*基因G312A多态位点与肺癌易感性关系的病例对照研究。对符合纳入标准的研究用*meta*分析的方法进行数据合并，采用RevMan 5.0和STATA 11.0评价研究间异质性，计算合并OR值及95%CI。并进行敏感性分析和发表偏倚检验。

**结果:**

共纳入18项研究，累计病例6 554例，对照8 322例。总体人群中A等位基因及AA基因型携带者肺癌风险明显升高（A *vs* G: OR=1.06, 95%CI: 1.00-1.12; AA *vs* AG+GG: OR=1.20, 95%CI: 1.06-1.36; AA *vs* GG: OR=1.19, 95%CI: 1.04-1.36）。亚洲人群中，AA基因型携带者肺癌风险明显升高（AA *vs* AG+GG: OR=7.15, 95%CI: 1.90-26.94; AA *vs* GG:OR=7.20, 95%CI: 1.91-27.15）。高加索人群中，AA基因型携带者肺癌风险升高（AA *vs* AG+GG: OR=1.15, 95%CI: 1.01-1.31）。

**结论:**

XPD 312A等位基因为肺癌发生的风险等位基因，AA基因型携带者肺癌风险升高，尤其在亚洲人群这种影响更为明显。

## Abstract (en)

**Background and objective:**

It has been proven that close relation was existed between *XPD* polymorphism G312A and lung cancer risk. However, some of the results are not consistent. The aim of this study is to explore the impact of DNA repair gene *XPD* polymorphism G312A on lung cancer risk.

**Methods:**

The literatures eligible from PUBMED, EMBASE, CNKI and WANGFANG database were enrolled in the *meta*-analysis. Heterogeneity among combined studies was assessed. The pooled OR and 95%CI were calculated. The sensitivity analysis and the publication bias were evaluated by Rev-Man 5.0 and STATA 11.0.

**Results:**

There were 6 554 cases and 8 322 controls from 18 studies included in the *meta*-analysis. In total, individuals with 312A allele and 312AA genotype showed increased lung cancer risk (A *vs* G: OR=1.06, 95%CI: 1.00-1.12; AA *vs* AG+GG: OR=1.20, 95%CI: 1.06-1.36; AA *vs* GG: OR=1.19, 95%CI: 1.04-1.36). In Asians, individuals with 312AA genotype showed 6.15 fold and 6.20 fold increased lung cancer risk in recessive genetic model and homogenous contrast respectively (AA *vs* AG+GG: OR=7.15, 95%CI: 1.90-26.94; AA *vs* GG: OR=7.20, 95%CI: 1.91-27.15). In Caucasians, individuals with 312AA genotype showed a 15% increased lung cancer risk (OR=1.15, 95%CI: 1.01-1.31).

**Conclusion:**

*XPD* 312A allele is risk allele for lung cancer. Individuals with AA genotype have higher risk of lung cancer, especially in Asians.

肺癌是对人类健康危害极大的一种疾病，在所有癌症中，肺癌导致死亡人数最多。大约31%与癌症相关的死亡是由肺癌造成的^[1]^。烟草使用是公认的肺癌最重要的危险因素，每年导致180万例癌症死亡（http://www.who.int）。烟草中包括许多致癌物和活性氧物质，它们导致产生DNA加合物、交叉联结、DNA损伤和DNA链断裂^[2]^。DNA修复系统对维持基因组的完整稳定和生物体的生存起着至关重要的作用。DNA修复缺陷是引起各种癌症的危险因素^[3]^。在机体的多种修复途径中，核苷酸切除修复（nucleotide excision repair, NER）是最灵活的DNA修复途径。NER可修复大块损伤比如紫外光造成的嘧啶二聚体、其它光产物、大的化学加合物和交叉联接物。烟草燃烧释放的多环芳烃类化合物诱导大块DNA加合物，主要由NER修复。XPD位于染色体19q13.2-13.3。作为ATP依赖的5’-3’解旋酶参与基础转录因子IIH（transcription factor IIH, TFIIH）复合体以行使分开双螺旋的功能。XPD蛋白对于正常的基础转录和NER都是必不可少的^[4]^。*XPD*基因的突变可能降低TFIIH复合体的活性，引起修复，转录缺陷和对于凋亡的异常反应^[5]^。

XPD G312A（Asp312Asn; reference SNP ID: rs1799793）是外显子10的一个G→A的转换，导致312密码子位置Asp→Asn的改变。是肺癌风险关联性研究中最常见的多态位点之一。最近研究^[6]^显示G312A多态明显降低健康个体淋巴细胞的XPD mRNA水平，并且在吸烟者和老年人群中更明显。宿主细胞激活实验^[7, 8]^显示，与野生型相比，312A等位基因变异与BPDE或紫外光诱导的DNA损伤的DNA修复能力降低有关。一些研究^[9-11]^报道XPD 312A等位基因变异与肺癌风险升高有关，而一项研究^[12]^显示G312A与肺癌风险无相关性。目前国内外有多篇XPD G312A多态位点与肺癌易感性的相关研究报道，但结果尚不一致。我们通过收集XPD G312A与肺癌易感性关系的病例对照研究，采用*meta*分析的方法以期对XPD G312A与肺癌易感性关系做出合理评价。

## 1 材料与方法

### 1.1 纳入文献的检索与筛选

检索PUBMED、EMBASE、清华CNKI全文数据库、万方全文数据库以得到关于XPD多态性与肺癌遗传易感性关系的所有病例-对照研究。检索截止时间为2010年2月。使用检索关键词及主题词为：XPD/ERCC2；lung cancer/neoplasm/tumor/carcinoma；polymorphism；肺癌。且无语种限制。对检索到的文献的参考文献也进行了检索。进行文献检索时由2位研究者分别进行检索，然后共同汇总。文献纳入符合以下标准：是独立的不相关的病例对照研究；研究中包括XPD G312A位点；对照组和病例组有明确的基因型频率分布数据；对研究间有共享样本的研究纳入其中样本量大的文献进入本研究；排除重复报告、数据不完整的文献进入本研究。

### 1.2 资料提取

对纳入研究的文献资料，提取以下内容并制成表 1。如果一篇文献中同时报道了两个以上不同人群的研究结果，我们将研究的每个人群看作为一个单独的研究。各研究的第一作者姓（名）、出版年代、研究对象种族、实施研究国家、对照组对象的来源、基因分型方法、病例组和对照组的人数及分型数据和对照组的次要等位基因频率。根据所纳入的文献，我们对种族来源进行了以下分类：高加索人、亚洲人、非洲裔美国人、拉丁裔和混合种族（文中受试者种族无明确说明）。

**1:** 纳入文献的一般情况（斜体字为不符合*HWE*的研究，MAF：次要等位基因频率） Characteristics of studies included in the *meta*-analysis (Italic type indicates the distribution of the genotypes in controls of the study is not in *HWE*. MAF: minor allele frequency)

First author (year)	Ethnicity	Country of research	Source of control	Genotyping	Lung cancer/Control	MAF of control
Hu (2006)^[15]^	Asian	China	Hospital	Taqman	970/986	A:0.057
Kong FJ (2006)^[26]^	Asian	China	Hospital	RFLP	120/120	A:0.058
Liang (2003)^[11]^	Asian	China	Population	RFLP	1 006/1 020	A:0.065
Shen (2005)^[16]^	Asian	USA	Population	Real-time PCR	118/113	A:0.062
*Yin (2008)^[17]^*	*Asian*	*China*	*Hospital*	*RFLP*	*201/171*	*A:0.006*
Butkiewicz (2001)^[10]^	Caucasian	Poland	Population	RFLP	96/94	A:0.436
De Ruyck (2007)^[18]^	Caucasian	Belgium	Hospital	RFLP	110/109	A:0.339
Hou (2002)^[9]^	Caucasian	Sweden	Population	RFLP	184/162	A:0.370
Lopez-Cima (2007)^[19]^	Caucasian	Spain	Hospital	RFLP	516/533	A:0.296
Matullo (2006)^[20]^	Caucasian	Italy	Population	Taqman	116/1 094	A:0.387
Misra (2003)^[12]^	Caucasian	Finland	Population	Taqman	313/312	A:0.364
Popanda (2004)^[21]^	Caucasian	Germany	Hospital	RFLP	463/460	A:0.370
Raaschou (2008)^[22]^	Caucasian	Denmark	Population	Real-time PCR	424/787	A:0.359
Zhou (2002)^[24]^	Caucasian	USA	Population	RFLP	1 092/1 240	A:0.331
Zienolddiny (2006)^[23]^	Caucasian	Norwegian	Population	Taqman	275/290	A:0.378
Chang (2008)^[25]^	African-American	USA	Population	GoldenGate	247/277	A:0.126
Chang (2008)^[25]^	Latinos	USA	Population	GoldenGate	108/297	A:0.197
Spitz (2001)^[7]^	Mixed-ethnicity	USA	Population	RFLP	195/257	A:0.272

### 1.3 统计学分析和资料合成

#### 1.3.1 研究效应测定指标

用比值比（odds ratio, OR）和95%可信区间（95%CI）作为每项研究结果的研究效应测定指标，衡量XPD G312A与肺癌风险之间关联的程度。对G312A位点，采用以下4种模型分析该位点与肺癌风险的关联。首先，以312AG+312GG两种基因型为对照，计算AA基因型与肺癌风险关联程度（隐性遗传效应模型）。再以312GG基因型为对照，计算携带突变等位基因A的基因型（312AG+312AA）与肺癌风险关联程度（显性遗传效应模型）。随后，以野生纯合基因型GG为对照，计算突变纯合基因型AA与肺癌风险关联程度。最后，以312G等位基因为对照，分析A等位基因和肺癌风险的关联程度。

#### 1.3.2 评价研究结果间异质性的指标及统计量的合并

采用两个指标*Q*值和*I*^2^值评价各研究结果间的异质性。基于χ^2^检验的*Q*检验^[13]^用于评估各研究间效应量分布是否具有异质性。以α=0.10为分界标准，当*P* > 0.1，表示各研究间效应量分布异质性不显著。采用固定效应模型（*Mantel-Haenszel*法）对各研究进行统计合并，当*P*≤0.1，采用随机效应模型（*Inverse-Variance*法）对各研究进行统计合并。用*Z*检验计算合并OR值的显著性（检验水准*P* < 0.05视为有统计学差异）。*I*^2^用于描述研究间变异占总变异的百分比。*I*^2^值在0-1之间。*I*^2^=0时研究间无异质性。*I*^2^趋近于0可能研究间的变异可能归因于偶然性。低于0.25则认为是轻度异质性。0.25-0.5之间为中度异质性。大于0.75则认为研究间有高度异质性^[14]^。针对种族进行亚组分层后进行效应值合并。对只有1项研究的亚组则不再进行效应值合并。

#### 1.3.3 发表偏倚的评价

根据漏斗图（funnel plots）肉眼观察其对称性以及*Begg*检验（*Begg’s* test）来判断纳入文章的发表偏倚。若漏斗图对称完整则无发表偏倚，否则可能有发表偏倚存在。采用发表偏倚检验时，若*P* < 0.05则认为存在发表偏倚。

#### 1.3.4 敏感性分析

用拟合优度χ^2^检验对纳入的每项研究的对照组的基因型频率分布进行检验，观察其是否符合*Hardy-Weinberg*平衡（*HWE*）。如果其不符合*HWE*，则该研究结果可能存在对象选择缺乏群体代表性或基因分型可能错误的现象。对不符合*HWE*的研究，观察其排除前后对*meta*分析结果有无影响以此进行敏感性分析（sensitivity analysis）并评估*meta*分析结果的强度及稳定性。

本研究分析采用STATA version 11.0（STATA Corporation）软件和Review Manager 5.0（Oxford, England）软件完成并绘制*meta*分析的森林图。所有P值都是双侧检验。

## 2 结果

### 2.1 纳入文献的一般情况

PUBMED、EMBASE、清华CNKI全文数据库和万方全文数据库的检索结果有17篇公开发表的文献（18项研究）符合纳入标准进入研究^[7, 9-12, 15-26]^。16篇文献为英文发表，1篇文献为中文发表。除了1项亚洲人群中的来自Yin等^[17]^的研究其对照组的基因型分布不符合*HWE*外，其他所有的研究其对照组的基因型分布都符合*HWE*。

总共累计病例6 554例，对照8 322例。本*meta*分析所纳入文献的细节见表 1，各研究病例组和对照组对象的等位基因频率及基因型分布见表 2。

**2:** 各研究病例组和对照组对象的等位基因频率及基因型分布 Distribution of G312A polymorphism genotype among cases and controls in the *meta*-analysis (Italic type indicates the distribution of the genotypes in controls of the study is not in *HWE*)

First author (year)	Ethnicity	Lung caner (*n*)				Control (*n*)		
		GG	AG	AA		GG	AG	AA
Butkiewicz (2001)^[10]^	Caucasian	43	35	18		29	48	17
Chang (2008)^[245]^	African American	186	58	3		212	60	5
Chang (2008)^[25]^	Latinos	60	40	8		192	93	12
De Ruyck (2007)^[18]^	Caucasian	44	53	13		49	46	14
Hou (2002)^[9]^	Caucasian	68	94	22		66	72	24
Hu (2006)^[15]^	Asian	850	116	4		874	111	1
Kong FJ (2006)^[26]^	Asian	101	17	2		106	14	0
Liang (2003)^[11]^	Asian	870	125	11		889	130	1
Lopez-Cima (2007)^[19]^	Caucasian	240	221	55		260	230	43
Matullo (2006)^[20]^	Caucasian	49	48	19		418	506	170
Misra (2003)^[12]^	Caucasian	143	127	43		125	147	40
Popanda (2004)^[21]^	Caucasian	182	211	70		192	196	72
Raaschou (2008)^[22]^	Caucasian	177	188	59		329	351	107
Shen (2005)^[16]^	Asian	109	9	0		99	14	0
Spitz (2001)^[7]^	mixed	102	72	21		135	104	18
Yin (2008)^[17]^	Asian	200	1	0		170	0	1
Zhou (2002)^[24]^	Caucasian	463	479	150		543	572	125
Zienolddiny (2006)^[23]^	Caucasian	119	102	54		120	121	49

### 2.2 *meta*分析结果

#### 2.2.1 总体分析结果

在总体人群，用A *vs* G、AA *vs* AG+GG、AA+AG *vs* GG、AA *vs* GG遗传模型分析G312A位点时，均没有发现研究间异质性存在。在固定效应模型下，A等位基因及AA基因型携带者肺癌风险明显升高（A *vs* G: OR=1.06, 95%CI: 1.00-1.12; AA *vs* AG+GG: OR=1.20, 95%CI: 1.06-1.36; AA *vs* GG: OR=1.19, 95%CI: 1.04-1.36）（图 1）。当排除不符合*HWE*的研究^[17]^后，以上结果无改变（表 3）。

**1:**
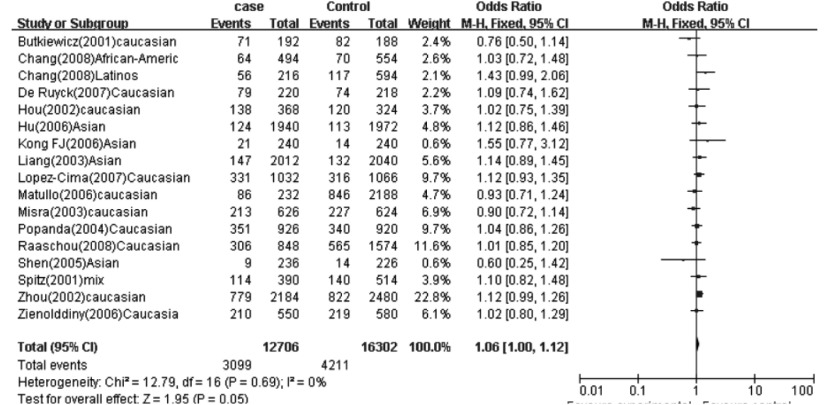
G312A位点总体分析结果（A *vs* G） *meta*-analysis for the XPD G312A polymorphism and lung cancer in total population (A *vs* G). Events=number of A allele, Total=number of A and C allele. ◆: pooled OR and its 95%CI

#### 2.2.2 亚组分析结果

在纳入的研究中，10项研究来自于高加索人群，5项研究来自于亚洲人群。我们分别对这两个人群进行了亚组分析。在亚洲人群中，在各遗传模型下，研究间无异质性存在，用固定效应模型合并效应量，结果显示，AA等位基因型携带者肺癌风险明显升高（AA *vs* AG+GG: OR=4.43, 95%CI: 1.51-12.99; AA *vs* GG: OR=4.45, 95%CI: 1.52-13.07）。当排除1项来自亚洲人群的不符合*HWE*的研究^[17]^后，在各遗传模型下，研究间仍然无异质性存在，用固定效应模型合并效应量，AA等位基因型携带者肺癌风险升高更加明显（AA *vs* AG+GG: OR=7.15, 95%CI: 1.90-26.94; AA *vs* GG:OR=7.20, 95%CI: 1.91-27.15）（图 2）。

**2:**
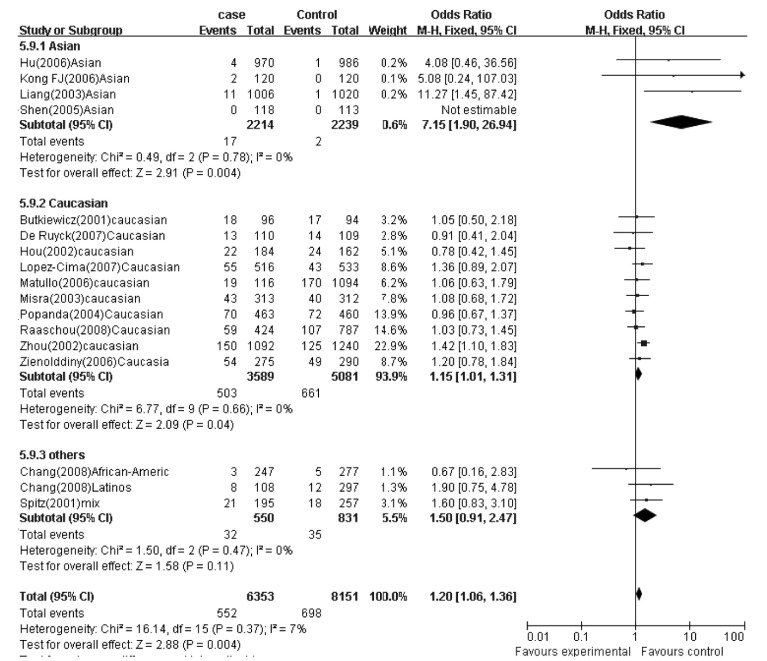
G312A位点在亚洲及高加索人群中的分析结果（AA *vs* AG+GG） *meta*-analysis for the XPD G312A polymorphism and lung cancer in Asians and Caucasians: AA *vs* AG+GG. Events=number of AA genotype, Total=number of individuals. ◆: pooled OR and its 95%CI

在高加索人群中，用各遗传模型分析G312A位点时，各研究间没有异质性存在。用固定效应模型合并效应量，结果显示，在AA *vs* AG+GG隐性遗传模型下，AA基因型携带者肺癌风险增加（OR=1.15, 95%CI: 1.01-1.31）（表 3）。

**3:** 不同遗传模型下XPD G312A位点的*meta*分析结果 Summary of OR with CI for various genetic contrasts of G312A polymorph and lung cancer risk (^*^the pooled analysis excluding the study that was not in *HWE*)

Ethnicity Studies		Contrast																		
	(Lung cancer/Control)	A *vs* G					AA *vs* AG+GG					AG+AA *vs* GG					AA *vs* GG			
		*P*_hetero_	*I*^2^	*P*	OR (95%CI)		*P*_hetero_	*I*^2^	*P*	OR (95%CI)		*P*_hetero_	*I*^2^	*P*	OR (95%CI)		*P*_hetero_	*I*^2^	*P*	OR (95%CI)
Total	18(6554/8322)	0.71	0	0.05	1.06(1.00-1.12)		0.39	5	0.004	1.20(1.06-1.36)		0.62	0	0.43	1.03 (0.96-1.11)		0.37	7	0.01	1.19(1.04-1.36)
Total^*^	17(6353/8151)	0.69	0	0.05	1.06(1.00-1.12)		0.37	7	0.004	1.20 (1.06-1.36)		0.55	0	0.42	1.03 (0.96-1.11)		0.35	9	0.01	1.19(1.04-1.36)
Asian	5(2415/2410)	0.48	0	0.20	1.12(0.94-1.32)		0.30	18	0.007	4.43(1.51-12.99)		0.65	0	0.47	1.07 (0.89-1.28)		0.30	17	0.007	4.45(1.52-13.07)
Asian^*^	4(2214/2239)	0.41	0	0.18	1.12(0.95-1.33)		0.78	0	0.004	7.15(1.90-26.94)		0.49	0	0.46	1.07 (0.89-1.28)		0.79	0	0.004	7.20(1.91-27.15)
Caucasian	10(3589/5081)	0.69	0	0.24	1.04 (0.97-1.11)		0.66	0	0.040	1.15 (1.01-1.31)		0.40	5	0.87	1.01 (0.92-1.10)		0.68	0	0.09	1.13 (0.98-1.30)

#### 2.2.3 敏感性分析

在本研究纳入的17篇文献共18项研究中，除了1项来自亚洲人群的研究其对照组的基因型频率分布不符合*HWE*外，其他研究均符合*HWE*。当对所有研究进行数据合并时，总体人群，亚洲人群，高加索人群研究间均无明显异质性存在。在固定效应模型下，对于总体人群，A等位基因及AA基因型携带者肺癌风险明显升高（A *vs* G: OR=1.06, 95%CI: 1.00-1.12; AA *vs* AG+GG: OR=1.20, 95%CI: 1.06-1.36; AA *vs* GG: OR=1.19, 95%CI: 1.04-1.36）。当排除不符合*HWE*的研究后，结果无改变。在亚洲人群，AA等位基因型携带者肺癌风险明显升高（AA *vs* AG+GG: OR=4.43, 95%CI: 1.51-12.99; AA *vs* GG: OR=4.45, 95%CI: 1.52-13.07）。当排除来自亚洲人群的不符合*HWE*的研究后，AA等位基因型携带者肺癌风险升高更加明显（AA *vs* AG+GG: OR=7.15, 95%CI: 1.90-26.94; AA *vs* GG: OR=7.20, 95%CI: 1.91-27.15）。

#### 2.2.4 发表偏倚

*Begg’s*漏斗图显示基本对称，纳入研究无明显发表偏倚。发表偏倚检验显示无发表偏倚存在（A *vs* G for XPD G312A: *t*=1.09, *P*=0.29）（图 3）。

**3:**
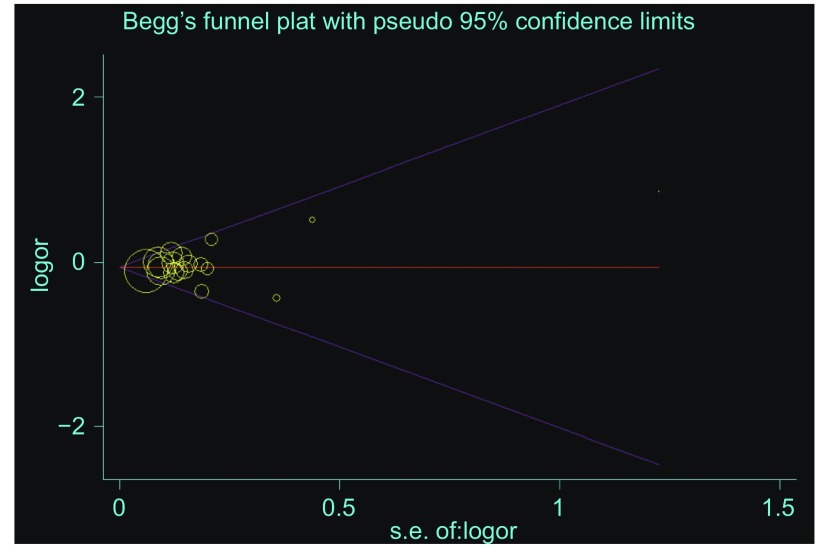
XPD G312A位点发表偏倚检验的*Begg’s*图（XPD G312A: A *vs* G） *Begg's* funnel plot for A *vs* G allele comparison in XPD G312A for publication bias

## 3 讨论

本研究对DNA修复基因*XPD* G312A与肺癌的18项病例-对照研究进行了*meta*分析。对纳入研究的所有文献进行数据合并，结果显示：对于总体人群，A等位基因及AA基因型携带者肺癌风险明显升高（A *vs* G: OR=1.06, 95%CI: 1.00-1.12; AA *vs* AG+GG: OR=1.20, 95%CI: 1.06-1.36; AA *vs* GG: OR=1.19, 95%CI: 1.04-1.36）。当排除不符合*HWE*的研究后，结果无改变。在亚洲人群，AA等位基因型携带者肺癌风险明显升高（AA *vs* AG+GG: OR=4.43, 95%CI: 1.51-12.99; AA *vs* GG: OR=4.45, 95%CI: 1.52-13.07）。当排除来自亚洲人群的不符合*HWE*的研究后，AA等位基因型携带者肺癌风险升高更加明显（AA *vs* AG+GG: OR=7.15, 95%CI: 1.90-26.94; AA *vs* GG: OR=7.20, 95%CI: 1.91-27.15）。在高加索人群中，在AA *vs* AG+GG隐性遗传模型下，AA基因型携带者肺癌风险增加（OR=1.15, 95%CI: 1.01-1.31）。功能性的研究^[7]^显示XPD G312A多态的DNA修复能力降低。312A等位基因变异基因型也与DNA加合物水平的增高有关^[9, 27]^。另外，变异的312A等位基因基因型与紫外光照射而不是X线照射后的染色体畸变（chromosome aberration, CA）增高明显相关，提示这些变异基因型可能在核苷酸切除修复途径存在缺陷^[28, 29]^。亚洲人群中，在隐性遗传模型及纯合子对照中AA等位基因携带者肺癌风险均增加3倍以上，而高加索人群中，只在隐性遗传模型分析时，AA等位基因携带者肺癌风险增加15%。人群不同的遗传背景可能是多态性与肺癌风险有不同关联性的原因。本研究显示，XPD G312A位点的变异等位基因的频率在高加索人群和亚洲人群有很大的不同。在纳入的研究中，亚洲人群312A变异等位基因频率均小于0.1。而高加索人群中报道的312A等位基因频率从0.296到0.436不等。远高于亚洲人群。两项*meta*分析^[30, 31]^考察了XPDG312A多态和癌或肺癌的关联。Kiyohara等^[31]^的研究发现312AA基因型和肺癌风险无明显关联。但另一项*meta*分析^[30]^报道在总体人群中312AA基因型和肺癌风险升高有关，与本研究一致。对于遗传关联研究的*meta*分析，是否纳入不符合*HWE*的研究尚无一致性的意见。Wang等^[30]^的研究排除了不符合*HWE*的研究，而Kiyohara等^[31]^纳入了所有的相关研究。Thakkinstian等^[32]^认为应该通过对排除或不排除符合*HWE*的研究进行数据合并来执行敏感性分析，以考察*meta*分析结果的强度和稳定性。本*meta*分析较Wang等^[30]^和Kiyohara等^[31]^的研究纳入了更多新的文献，未发现发表偏倚存在，基于种族背景考察了更多的细节，并进行了敏感性分析以评估我们结果的强度和稳定性。我们发现排除不符合*HWE*的研究后，XPD G312A位点的*meta*分析的结果均未改变。

综上，本研究认为XPD G312A多态可影响个体肺癌发生的风险，尤其在亚洲人群这种影响更为明显。应该实施更多设计严谨、样本量大的研究考察不同人群中该位点与肺癌发病的关联。
